# Association Between Nitric Oxide, Oxidative Stress, Eryptosis, Red Blood Cell Microparticles, and Vascular Function in Sickle Cell Anemia

**DOI:** 10.3389/fimmu.2020.551441

**Published:** 2020-11-04

**Authors:** Elie Nader, Marc Romana, Nicolas Guillot, Romain Fort, Emeric Stauffer, Nathalie Lemonne, Yohann Garnier, Sarah Chambers Skinner, Maryse Etienne-Julan, Mélanie Robert, Alexandra Gauthier, Giovanna Cannas, Sophie Antoine-Jonville, Benoît Tressières, Marie-Dominique Hardy-Dessources, Yves Bertrand, Cyril Martin, Céline Renoux, Philippe Joly, Marijke Grau, Philippe Connes

**Affiliations:** ^1^ Laboratoire Interuniversitaire de Biologie de la Motricité (LIBM) EA7424, Team « Vascular Biology and Red Blood Cell », Université Claude Bernard Lyon 1, Université de Lyon, Lyon, France; ^2^ Laboratoire d’Excellence du Globule Rouge (Labex GR-Ex), PRES Sorbonne, Paris, France; ^3^ Université des Antilles, Pointe-à-Pitre, France; ^4^ Université de Paris, Paris, France; ^5^ Laboratoire Carmen Inserm, Université Claude Bernard Lyon 1, Université de Lyon, Villeurbanne, France; ^6^ Département de Médecine Interne, Hôpital Edouard Herriot, Hospices Civils de Lyon, Lyon, France; ^7^ Centre de Médecine du Sommeil et des Maladies Respiratoires, Hospices Civils de Lyon, Hôpital de la Croix Rousse, Lyon, France; ^8^ Unité Transversale de la Drépanocytose, Hôpital de Pointe-á-Pitre, Hôpital Ricou, Guadeloupe, France; ^9^ Erytech Pharma, Lyon, France; ^10^ Institut d’Hématologie et d’Oncologie Pédiatrique, Hospices Civils de Lyon, Lyon, France; ^11^ Centre Investigation Clinique Antilles Guyane, 1424 Inserm, Academic Hospital of Pointe-á-Pitre, Pointe-á-Pitre, Guadeloupe, France; ^12^ Laboratoire de Biochimie et de Biologie Moléculaire, UF de Biochimie des Pathologies érythrocytaires, Centre de Biologie et de Pathologie Est, Hospices Civils de Lyon, Lyon, France; ^13^ Molecular and Cellular Sport Medicine, Deutsche Sporthochschule Köln, Köln, Germany; ^14^ Institut Universitaire de France, Paris, France

**Keywords:** sickle cell anemia, eryptosis, red blood cell microparticles, vascular dysfunction, endothelial cells, TLR4

## Abstract

Chronic hemolysis, enhanced oxidative stress, and decreased nitric oxide (NO) bioavailability promote vasculopathy in sickle cell anemia (SCA). Oxidative stress and NO are known to modulate eryptosis in healthy red blood cells (RBCs); however, their role in SCA eryptosis and their impact on the genesis of RBC-derived microparticles (RBC-MPs) remains poorly described. RBC-MPs could play a role in vascular dysfunction in SCA. The aims of this study were to evaluate the roles of oxidative stress and NO in eryptosis and RBC-MPs release, and to determine whether RBC-MPs could be involved in vascular dysfunction in SCA. Markers of eryptosis and oxidative stress, plasma RBC-MPs concentration and arterial stiffness were compared between SCA and healthy (AA) individuals*. In-vitro* experiments were performed to test: 1) the effects of oxidative stress (antioxidant: n-acetylcysteine (NAC); pro-oxidant: cumene hydroperoxide) and NO (NO donor: sodium nitroprusside (SNP); NO-synthase inhibitor (L-NIO)) on eryptosis, RBC deformability and RBC-MP genesis; 2) the effects of SCA/AA-RBC-MPs on human aortic endothelial cell (HAEC) inflammatory phenotype and TLR4 pathway. Eryptosis, RBC-MPs, oxidative stress and arterial stiffness were increased in SCA. NAC increased RBC deformability and decreased eryptosis and RBC-MPs release, while cumene did the opposite. SNP increased RBC deformability and limited eryptosis, but had no effect on RBC-MPs. L-NIO did not affect these parameters. Arterial stiffness was correlated with RBC-MPs concentration in SCA. RBC-MPs isolated directly from SCA blood increased adhesion molecules expression and the production of cytokines by HAEC compared to those isolated from AA blood. TLR4 inhibition alleviated these effects. Our data show that oxidative stress could promote eryptosis and the release of RBC-MPs that are potentially involved in macrovascular dysfunction in SCA.

## Introduction

Sickle cell anemia (SCA) is the most prevalent genetic disease worldwide. SCA is caused by a mutation in the β-globin gene that leads to the substitution of a glutamic acid by a valine at the 7^th^ codon, which results in the production of abnormal hemoglobin called hemoglobin S (HbS). HbS can polymerize in deoxygenated conditions, causing red blood cell (RBC) sickling. Sickled RBCs are poorly deformable and very fragile. Indeed, patients with SCA suffer from chronic hemolytic anemia and repeated vaso-occlusive crises (1). Accumulating evidence suggests that chronic hemolysis, enhanced oxidative stress and decreased bioavailability of nitric oxide (NO) are at the origin of the vascular dysfunction observed in patients with SCA (2–4), which could be involved in the pathogenesis of several acute and chronic complications (3). In addition, heme released by red blood cells into the plasma could play a role in vascular dysfunction by promoting inflammation through the activation of Toll Like Receptor 4 (TLR4) (5).

Furthermore, it has been shown that oxidative stress could promote eryptosis, i.e., RBC suicidal death, in healthy RBCs *in vitro* (6). A previous study has also suggested that NO prevents eryptosis induced by calcium ionophore stimulation (7). Eryptosis is characterized by increased intra-erythrocyte calcium levels, increased phosphatidylserine (PS) exposure, RBC shrinkage (followed by a decrease of RBC deformability), energetic depletion and membrane blebbing (8). Eryptosis has been shown to be increased in SCA (8). Several authors reported alteration in the PS asymmetry of the RBC membrane (9) and high RBC calcium levels in SCA (10–12) but the exact role of oxidative stress and NO in eryptosis in SCA is unknown.

RBC membrane blebbing during eryptosis could lead to the release of microparticles (MPs) into the blood circulation (8, 13). Several studies observed increased levels of MPs in the blood of SCA patients compared to healthy individuals at steady state (14, 15), and a further rise during vaso-occlusive crisis (16, 17), with platelet- and RBC-derived MPs (RBC-MPs) representing the majority of the circulating MPs detected (18). It has been suggested that RBC-MPs may promote vascular dysfunction in SCA. A recent study by Camus et al. (19) showed that SCA RBC-MPs could promote the apoptosis of endothelial cells *in vitro*, and stimulate renal vaso-occlusion in a sickle cell mice model. However, the MPs used in this study were artificially generated *ex vivo*, and were not representative of RBC-MP properties *in vivo*. Furthermore, the mechanisms at the origin of the enhanced RBC-MPs release, and especially the implications of high oxidative stress and decreased NO bioavailability, are still poorly understood in SCA.

The primary aims of our study were to investigate the effects of oxidative stress and NO on eryptosis and the release of MPs by sickle RBCs, and to explore the role of RBC-MPs generated *in vivo*, and thus isolated from SCA patients, in the vascular dysfunction associated with SCA. We compared several markers of eryptosis and RBC-MPs plasma concentrations between healthy individuals and patients with SCA, and then modulated these biological parameters *in vitro* using agents that modify oxidative stress and NO bioavailability. *In vivo* macrovascular function was also evaluated in patients, and activation of endothelial cells from the macrocirculation were evaluated after *in vitro* incubation with RBC-MPs directly isolated from blood of AA individuals and SCA patients, with and without a TLR4 inhibitor.

## Methods

### Subjects and Sampling

A total of sixty-two patients with SCA (SS, 25 children, 37 adults) and 22 healthy controls (AA, 10 children, 12 adults) were included in the different experiments (see **Table 1** for subject characteristics). Patients with SCA were recruited from the Edouard Herriot Hospital (Lyon, France), the Institut d’Hématologie et d’Oncologie Pédiatrique (Lyon, France) and the University hospital of Pointe à Pitre (Guadeloupe, France). All subjects were in clinical steady-state at the time of the study; i.e., without any vaso-occlusive crisis or other acute medical complication within the last 2 months, and without any blood transfusions for at least 3 months before inclusion. Patients received common treatment as recommended in sickle cell disease (vitamin B9 and vitamin D). The mean daily dose of hydroxyurea for patients receiving this therapy (n = 55/62) was 18.8 ± 0.9 mg/kg. The study was conducted in accordance with the guidelines set by the Declaration of Helsinki, and all subjects gave informed written consent before their participation. The study was approved by the CPP Sud-Est IV (Lyon, France, L16-47) and the CPP Sud/Ouest Outre Mer III (Bordeaux, France, 2012‐A00701‐42) Ethics Committees.

**Table 1:** Hematological characteristics of subjects and patients included.

	AA	SCA
N [HU+]	22	62 [55]
Age (yrs)	21.5 ± 2.2	24.5 ± 2.1
Hb (g/dl)	/	8.5 ± 0.4
HbF (%)	/	15.7 ± 8.4
MCV (fl)	/	95 ± 13.1

AA, healthy subjects; SCA, sickle cell anemia patients; N, number; HU+, patients under hydroxyurea treatment; yrs, years; Hb, hemoglobin; HbF, fetal hemoglobin; MCV, Mean Corpuscular Volume.

Data are presented as mean ± SD.

Venous blood was taken from the antecubital vein and collected into EDTA tubes for RBC deformability and hematological measurements, and into citrate tubes for eryptosis and microparticle analyses (BD Vacutainer, Plymouth, UK). Serum lactate dehydrogenase (LDH) concentration was determined by standard biochemical methods.

### Arterial Stiffness

Arterial stiffness is considered to be a relevant indicator of macrovascular function (20), and is increased in patients with SCA (21). We evaluated arterial stiffness by measuring carotid-radial pulse wave velocity (CR-PWV) with a non-invasive automated device (SphygmoCor System, Actor, Sydney, Australia). A 3-lead electrocardiogram was used and pressure waves were recorded using an arterial tonometer. Pulse wave velocity (PWV) was calculated as the distance between two measuring sites, divided by the transit time (in seconds) of the related pulse waves. Transit time was defined as the difference between the delay of the distal pulse wave to the R wave of the ECG and the delay of the proximal pulse wave to R wave of the ECG. The pulse wave delay was determined by calculating the time elapsed from the peak of the R wave and the foot of the pressure pulse wave. The same experienced operator conducted the measurements throughout the whole study. Measurements were repeated two times at each measurement site, and the mean values were calculated and used for analyses. This technique has been demonstrated to be highly reproducible in both healthy and diseased populations (22, 23), and has been previously been used in individuals with SCA (21, 24).

### RBC Deformability

RBC deformability was assessed at 37°C, at 3 and 30 Pa by laser diffraction analysis (ektacytometry), using the Laser-assisted Optical Rotational Cell Analyzer (LORRCA MaxSis, RR Mechatronics, Hoorn, The Netherlands). The system has been described in detail elsewhere (25). Briefly, 10 μl of blood were mixed with 1 ml polyvinylpyrrolidone (PVP; viscosity ≈ 30 cP) and sheared into a Couette system. A laser beam was directed through the samples. The shear stress-induced deformation of RBCs affected the laser beam’s diffraction pattern, which was measured by the LORRCA software, and used to calculate an elongation index. A higher elongation index represents greater RBC deformability. The procedure was carried out according to the international methodological recommendations (25, 26).

For pharmacological modulation experiments, whole blood was centrifuged (800*g*, 10 min at 20°C), and plasma and buffy coat were discarded. RBCs were washed in PBS 1× buffer, and the RBC pellets were resuspended at a hematocrit (Hct) of 20% in PBS with either sodium nitroprusside (SNP; 100 µM), a NO donor, L-NIO (10 µM), a nitric oxide synthase (NOS) inhibitor, n-acetylcysteine (NAC; 2,5 mM), an antioxidant agent, or cumene hydroperoxide (100 µM), a pro-oxidant molecule, or vehicle for the control condition, and then incubated for 40 min at 37°C. RBC suspensions were then washed with PBS and resuspended in PBS buffer (Hct = 20%), and RBC deformability was measured as previously described.

### Measurement of RBC Nitrite

Previous studies have shown that nitrite levels reflect NO content in both physiological and pathophysiological conditions (27, 28). RBC nitrite content in control condition and after incubation with SNP was measured in order to confirm the effectiveness of the NO donor properties of the molecule within RBCs. Measurements of RBC nitrite content were realized according to previous studies (29, 30).

For nitrite measurement in RBCs, methanol (VWR international, Darmstadt, Germany) was added to the RBC frozen samples in a 1:2-ratio to remove proteins and the suspensions were centrifuged at 21 000 g, at 4°C for 15 min to collect the supernatants.

An ozone-based chemiluminescence NO detector was used to determine Nitrite levels (CLD 88e, EcoPhysics, Switzerland). Samples were injected into an acidified tri-iodide solution that reduces nitrite but also iron-nitrosylheme, and S-nitrosothiols to NO gas. NO is transported by a helium gas stream to a NaOH trap and finally transported to the CLD device where it can be measured by its gas-phase chemiluminescent reaction with ozone. The tri-iodide solution stoichiometrically releases NO from nitrite. Samples were measured in triplicate. A calibration curve with solution containing known concentrations of nitrite was realized to calculate nitrite concentration in the samples.

Nitrite content of methanol was also determined and nitrite concentrations measured in RBC samples were adjusted accordingly. Data analysis was done with the Chart FIA software (Ecophysics, Switzerland) to integrate the area under the curve.

### RBC β-Spectrin S-Nitrosylation

NO has been shown to S-nitrosylate cytoskeletal protein β-spectrin (28). S-nitrosylation of the β-spectrin in SCA patients in control and SNP conditions was assessed using the S-Nitrosylated Protein Detection Assay kit (Cayman Chemical, Ann Arbor, MI, USA) according to manufacturer’s instructions. The protocol contains three steps: 1) blocking of free SH groups; 2) cleavage of potential S-NO bonds and 3) biotinylation and avidin labeling of the newly formed SH groups. Additionally, the protein concentration of the samples was determined using the DC-Protein Assay Kit (BioRad, Munich, Germany) to ensure that equal amounts of protein were analyzed. A total of 20 μg protein was loaded into each lane of a 3–8% Tris-acetate gel (BioRad) and separated for 1 h under constant 90 mA current in a 1 × XT Tricine running buffer (BioRad). Proteins were blotted onto a polyvinylidene difluoride (PVDF) membrane (0.45 mm pore size). Background was blocked in 2% bovine serum albumin (in 1× TBS with 0.1% Tween 20) overnight at room temperature. Horseradish peroxidase (dilution 1:2000) was added which binds to the biotin-avidin complex, and the reaction was developed using a chemiluminescence kit containing peroxidase substrate (Thermo Fischer Scientific, Darmstadt, Germany). S-nitrosylated protein band of 220 kDa, previously identified as β-spectrin, was analyzed for differing “Integrated densities” using “Image J” software.

### Systemic Oxidative Stress Markers

Plasma Advanced Oxidation Protein Products (AOPP) were determined using the semi-automated method developed by Witko-Sarsat et al. (31), as previously described (32). Concentrations of plasma MDA were determined as previously described (32), using a modified method reported by Ohkawa et al. (33), based on thiobarbituric acid reactions.

### Eryptosis Markers

#### RBC Preparation

Citrate tubes were centrifuged (800 g, 10 min at 20°C) and plasma and buffy coat were discarded. RBCs were washed in PBS 1×, and RBC pellets resuspended at 0.4% Hct in PBS buffer containing 2.5 mM Ca^2+^ (for PS, ROS, and Ca^2+^ analysis) or 5 mM EDTA (for the PS negative control). For pharmacological modulation experiments, suspensions were incubated at 37°C for 40 min with either SNP (100 µM), L-NIO (10 µm), NAC (2.5 mM), cumene hydroperoxide (100 µM) or the vehicle for the control condition. Then, RBC suspensions were washed with PBS 1× and resuspended in PBS buffer as mentioned above.

#### Phosphatidylserine Exposure

PS exposure on the outer membrane leaflet of the RBCs was evaluated by using Annexin V-FITC, which binds to PS. RBC suspensions were protected from light and incubated for 30 min at room temperature (RT) with Annexin V-FITC (1:200 dilution, Beckman Coulter, California, US). Immediately after incubation, samples were diluted and analyzed by FACS (BD Accuri C6, Franklin Lakes, USA). PS exposure was measured in the FITC channel (with an excitation wavelength of 488 nm and an emission wavelength of 530 nm) according to manufacturer’s instructions. Negative controls were obtained by replacing Ca^2+^ by EDTA to prevent Annexin V from binding to PS. For each sample, 50 000 events, gated for the appropriate Forward Scatter (FSC), were counted.

#### Reactive Oxygen Species (ROS)

Intracellular RBC oxidative stress was determined using 2′,7′–dichlorofluorescin diacetate (DCFDA, Sigma-Aldrich, Saint-Quentin-Fallavier, France). RBC suspensions at 0.4% Hct were incubated for 30 min at RT in the dark with 10 μM of DCFDA (Sigma-Aldrich, Saint-Quentin-Fallavier, France). The samples were then analyzed using FACS, according to manufacturer’s instructions. The Median Fluorescence Intensity (MFI) of 50 000 gated events was recorded to quantify ROS levels.

#### Intracellular Calcium (Ca^2+^)

RBC Ca^2+^ content was measured with a Fluo3/AM (Biotium, Fremont, USA) probe. RBC suspensions were incubated for 30 min at RT with 5 μM of Fluo3/AM, and analyzed using FACS, according to the manufacturer’s instructions. MFI of the 50 000 gated events was recorded to quantify Ca^2+^ levels.

#### Glucose Uptake

RBC glucose uptake was analyzed using the 2-NBD-Glucose (Abcam, Cambridge, USA) probe. RBC suspensions were incubated for 30 min at RT with 200 µM of 2-NBD-Glucose, and analyzed by FACS according to manufacturer’s instructions. The MFI of the 50 000 gated events was recorded to quantify levels of glucose uptake.

### RBC Microparticles Extraction and Quantification

Microparticles were quantified as previously reported (34). Briefly, citrate tubes were centrifuged at 1 000 g for 10 min at 20°C. Platelet poor plasma was then submitted to ultracentrifugation (20 000 g, 20 min at 20°C) to extract MPs. Supernatant was discarded and the pellet was washed twice in working buffer (10 mM HEPES pH 7.4, 136 mM NaCl, 5 mM KCl, 2 mM MgCl_2_) containing 5 mM of EDTA for the first washing step and no EDTA for the second one. Working buffer was finally added to the MPs pellet, and the suspensions were stored at −80°C until the day of analysis. MPs quantification was performed with the FC500 Beckman Coulter flow cytometer (Beckman Coulter, Brea, CA, USA) and calibrated fluorescent microbeads (Flowcount; Beckman Coulter). MPs were incubated with Annexin V-FITC and an anti-CD235a-PE antibody to specifically quantify MPs from RBCs. The Megamix kit was used to standardize MPs acquisition gate based on fluorescent microbead size (0.5, 0.9, and 3 μm; Biocytex, Marseille, France) according to the supplier’s instructions. MPs were defined as events both smaller or equal in size to the 0.9 µm-large microbeads, and positively labeled with Annexin V-FITC.

For pharmacological modulation experiments, whole blood collected in citrate tube was centrifuged (800*g*, 10 min at 20°C), and plasma and buffy coat were discarded. RBCs were washed in PBS, and RBC pellets were resuspended at 20% Hct in PBS buffer containing 2.5 mM Ca^2+^ with either SNP (100 µM), L-NIO (10 µM), NAC (2.5 mM), cumene hydroperoxyde (100 µM) or vehicle for control condition for 24 h at 37°C and under constant shaking. Then, microparticles were extracted in the supernatant and quantified as previously described.

### Red Blood Cell Microparticle Isolation

Red blood cell microparticles were negatively selected from total purified MPs by using a depletion kit composed of anti-PE microbeads and LD columns (130-097-054 and 130-042-901, both from Macs, Miltenyi Biotec, Bergisch Gladbach, Germany) according to the manufacturer’s instructions. Briefly, total MPs were incubated with relevant PE conjugated antibodies targeting unwanted MPs (anti-CD15, anti-CD41, anti-CD14, and anti-CD106 antibodies). Then, microbeads coupled with antibody directed against PE were added to bind to these antibodies and allowed subsequent depletion of unwanted MPs by applying a strong magnetic field. FACs was used to quantify purified RBC-MPs using the method previously described. The depletion procedure allowed to obtain a suspension containing 95% of RBC-MPs (see **Figure 1** in **Supplementary Data**).

### Endothelial Cell Incubation With Sickle RBC-Derived Microparticles and Cytokine Supernatant Analysis

Human aortic endothelial cells (HAEC, Promocell Germany) were grown at 90% confluence in 96 well plates with fetal bovine serum (MV2 medium). Cells were pre-activated with TNF-α (0.6 ng/ml), as previously described (35, 36). Then, RBC-MPs that had been directly isolated from the plasma of SCA patients and healthy individuals, were incubated with HAEC cells for 24 h at a concentration of 50 000 MPs/100 µl with or without TAK 242 (2 µM), a TLR4 inhibitor. For negative controls, cells were incubated with TAK 242 vehicle (DMSO). Then, cells were washed twice with PBS containing BSA, detached from wells with Accutase (StemPro Accutase, Thermofisher Scientific) and resuspended in PBS-BSA. Cells were stained for 20 min at RT with anti-CD62E-FITC (0.125 µg/100 µl) and anti-CD54-PE (0.125 µg/100 µl) antibodies to assess E-Selectin and ICAM-1 expression at the surface of the cells. Stained cells were washed and re-suspended in FACS buffer, and analyzed by flow cytometry, according to manufacturer’s instructions. MFI or percentage of positive cells were recorded to quantify E-Selectin and I-CAM1 expression.

Cell supernatant in the wells was immediately collected after the 24-h incubation, and stored at −80°C until analysis. Cytokines and stimulating factors in the supernatant were quantified by Bio-Plex Multiplex immunoassay (Biorad, California, USA), using the Bio-Plex Pro™ Human Cytokine 17-plex Assay kit and the BioPlex 3D platform (Biorad, California, USA), according to manufacturer’s instructions.

### Statistical Analysis

Data are represented as individual points with mean. Comparisons between AA and SCA groups were achieved by using student T-tests or Mann-Whitney tests, when appropriate. The effects of molecules on SCA RBCs were analyzed by using paired t-tests and a Friedman test for RBC-MPs production modulation. Pearson or Spearman correlations were performed to test the associations between the parameters investigated. Multivariate linear regression analysis was performed to test the presence of independent associations. Effects of RBC-MPs on endothelial cells were analyzed using Friedmann test followed by Dunn’s *post hoc* test for multiple comparisons. GraphPad Prism 7 (La Jolla, CA, USA) and SPSS 23.0 (IBM, Armonk, NY, USA) softwares were used for statistical analyses. A p-value < 0.05 was considered as significant.

## Results

### Eryptosis and RBC-MPs Are Increased in SCA

**Figure 1:**
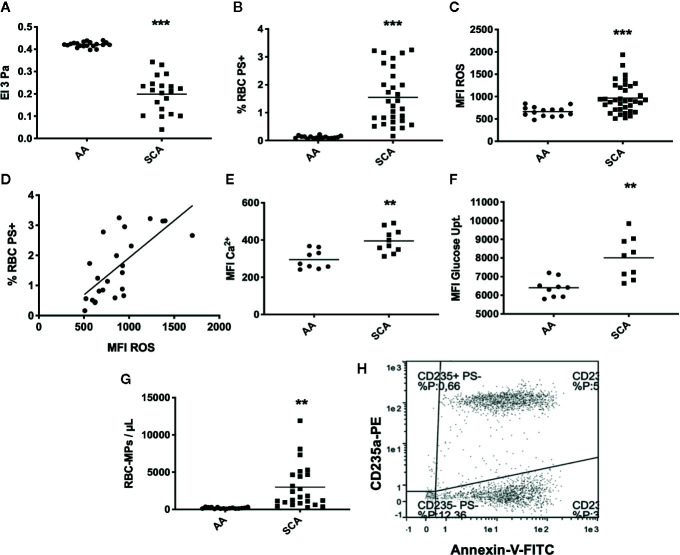
RBC deformability, eryptosis markers, and RBC-MPs in SCA and healthy (AA) individuals. **(A)** RBC deformability in SCA (n = 35) and AA (n = 20) subjects. **(B)** RBC PS exposure in SCA (n = 30) and AA (n = 20) subjects. **(C)** ROS content in SCA (n = 31) and AA (n = 20) subjects. **(D)** Correlation between PS exposure and ROS level in SCA (correlation was made in 23 patients who had both RBC-PS exposure and RBC ROS measurements, using Pearson correlation). **(E)** RBC Ca^2+^ level in SCA (n = 10) and AA (n = 9) subjects. **(F)** Glucose uptake in SCA (n = 9) and AA (n = 9) subjects. **(G)** RBC-MP plasma concentrations in SCA (n = 24) and AA (n = 16) subjects. **(H)** Representative flow-cytometric dot-plot used to quantify RBC-MPs in SCA patient. RBC-MPs were determined as events smaller than 0.9 µm and both positive for annexin-V and CD235a. Significant difference between AA and SCA. **p < 0.01, ***p < 0.001. Statistical comparisons between AA and SCA for RBC deformability, PS exposure, ROS content and MPs concentration were achieved using Student T-test. Statistical comparisons between AA and SCA for Ca^2+^ level and glucose uptake content were achieved using Mann Whitney test. MFI, mean fluorescence intensity; %RBC PS+, percentage of RBCs positive for Annexin-V; ROS, level of reactive oxygen species in RBCs detected with DCFDA; Ca2+, level of calcium in RBCs detected with Fluo3.

As expected, RBC deformability was lower in SCA patients than in healthy individuals (p < 0.001, **Figure 1A**). Analysis of eryptosis markers by FACS revealed that all markers were significantly increased in SCA RBCs compared to healthy RBCs: PS externalization at the surface of SCA RBCs (p < 0.001, **Figure 1B**, see **Figures 2A, B** in **Supplementary Data** for representative dot plots), RBC ROS content (p < 0.001, **Figure 1C**), intracellular levels of Ca^2+^ (p < 0.01, **Figure 1E**) and glucose uptake (p < 0.05, **Figure 1F**). ROS levels and the percentage of RBCs with externalized PS were positively correlated (p < 0.0001, r = 0.70, **Figure 1D**). RBC-MPs concentration was increased in SCA patients compared to healthy individuals (p < 0.01, **Figure 1G**). **Figure 1H** represents a flow-cytometric dot-plot image used to specifically identify RBC-MPs.

### NO and Oxidative Stress Modulate RBC Deformability, Eryptosis Markers, and MPs Emission by Sickle RBCs

**Figure 2:**
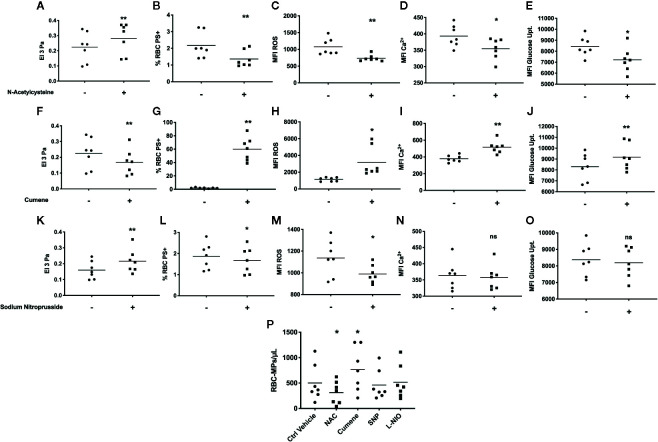
*In vitro* modulation of RBC deformability, eryptosis, and RBC-MPs by oxidative stress and NO related agents on SCA RBCs. Impact of NAC (n = 7) on SCA RBC deformability **(A)**, RBC PS exposure **(B)**, ROS level **(C)**, RBC Ca^2+^ content **(D)** and glucose uptake **(E)**. Effect of cumene hydroperoxide (n = 7) on SCA RBC deformability **(F)**, RBC PS exposure **(G)**, ROS level **(H)**, RBC Ca^2+^ content **(I)** and glucose uptake **(J)**. Effect of SNP (n = 7) on SCA RBC deformability **(K)**, RBC PS exposure **(L)**, ROS level **(M)**, RBC Ca^2+^ content **(N)** and glucose uptake **(O).** Impact of NAC, cumene hydroperoxide, SNP and L-NIO on the release of MPs by RBCs in the supernatant **(P)** (n = 7). Significantly different from the control condition: *p < 0.05, **p < 0.01. ns, not significant. All statistical comparisons were performed using paired T-test except for the MPs modulation where a Friedmann test was used. MFI, mean fluorescence intensity; %RBC PS+, percentage of RBCs positive to Annexin-V; ROS, level of reactive oxygen species in RBCs detected with DCFDA; Ca2+, level of calcium in RBCs detected with Fluo3; NAC: N-Acetylcysteine; SNP, sodium nitroprusside; Cumene, cumene hydroperoxide; −, incubation with ctrl vehicle, +, incubation with pharmacological molecule.

Incubation of SCA RBCs with the anti-oxidant agent NAC caused an increase of RBC deformability (p < 0.01, **Figure 2A**) and a decrease of the percentage of PS-exposing RBCs (p < 0.01, **Figure 2B**), RBC ROS content (p < 0.01, **Figure 2C**), RBC Ca^2+^ content (p < 0.05, **Figure 2D**) and RBC glucose uptake (p < 0.05, **Figure 2E**). The experiments performed with cumene hydroperoxide showed a decrease of RBC deformability (p < 0.01, **Figure 2F**), and an increase of the percentage of PS-exposing RBCs (p < 0.01, **Figure 2G**), RBC ROS content (p < 0.05, **Figure 2H**), RBC Ca^2+^ content (p < 0.01, **Figure 2I**), and RBC glucose uptake (p < 0.05, **Figure 2J**).

The use of the NO donor SNP also led to a rise of RBC deformability (p < 0.01, **Figure 2K**), a decrease of the percentage of PS-exposing RBCs (p < 0.05, **Figure 2L**) and RBC ROS content (p < 0.05, **Figure 2M**), but no significant change was observed for RBC Ca^2+^ content (**Figure 2N**) or RBC glucose uptake (**Figure 2O**). SNP also significantly increased RBC nitrite content and RBC β-Spectrin S-nitrosylation (see **Figures 3A, B** in **Supplementary Data**). The NOS inhibitor L-NIO had no significant effect on RBC nitrite content, deformability and eryptosis markers (data not shown).

SCA RBCs were also incubated for 24 h with NAC, cumene, SNP and L-NIO to test the effects of these chemical agents on the release of MPs. The results (**Figure 2P**) demonstrated that NAC decreased the amount of RBC-MPs (p < 0.05) while cumene hydroperoxide increased their release (p < 0.05). No significant effect of SNP and L-NIO was observed.

**Figure 3:**
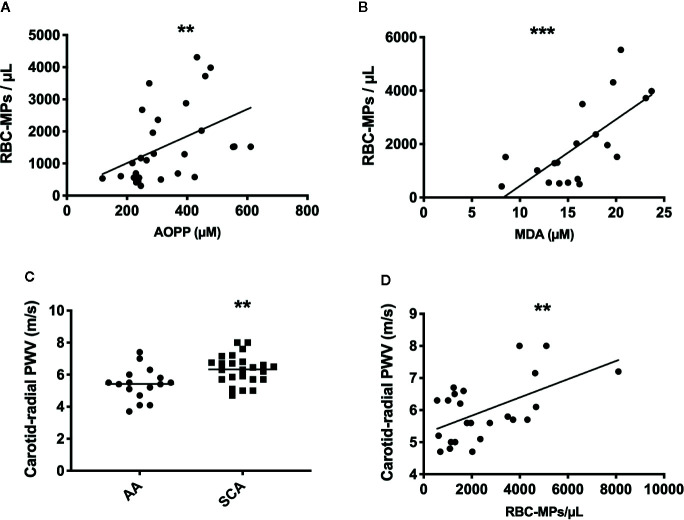
Relationship betweent RBC-MPs, oxidative stress and carotid-radial pulse wave velocity (PWV) in SCA patients, and comparaison of PWV between AA and SCA individuals. Correlation between RBC-MPs plasma concentration and AOPP (n = 28) **(A)** and MDA (n = 19) **(B)** concentration in SCA patients. Carotid-radial PWV measured in 24 SCA and 16 AA subjects **(C)**. Correlation between RBC-MPs plasma concentration and carotid-radial PWV in 24 SCA patients **(D)**. **p < 0.01, ***p < 0.001. Statistical correlations were determined using Pearson correlation test. Comparison between PWV in SCA and AA individuals were performed using Student T-test. PWV, pulse wave velocity.

Following the quantification of RBC-MPs, AOPP and MDA levels in the blood of a SCA cohort at steady state (n = 28), positive correlations between several parameters were observed (RBC-MPs vs AOPP: r = 0.44, p < 0.01; RBC-MPs vs MDA: r = 0.66, p < 0.001; **Figures 3A, B**, respectively). These results suggest that the higher the systemic oxidative stress, the higher the amount of circulating RBC-derived MPs.

### Arterial Stiffness Is Increased in SCA Patients and Correlates With the Amount of Circulating RBC-MPs

CR-PWV was measured in a cohort of 24 SCA patients, and compared to a group of AA (n = 16) of the same ethnic origin. As previously reported (21), PWV was higher in SCA than in AA (**Figure 3C**, p < 0.01), indicating increased arterial stiffness in the former group. Circulating RBC-MPs were also measured in 24 patients of this cohort to test the association with the carotid-radial PWV. Our results showed a positive association between these two parameters (**Figure 3D**, p < 0.01, r = 0.55). Because chronic anemia may lead to important cardiovascular adaptations in order to compensate for the decrease in hemoglobin concentration (37), and because chronic hemolysis participates in the development of vasculopathy in SCA (38), a multivariate linear analysis was performed between carotid-radial PWV and RBC-MPs, LDH, AOPP, MDA and hemoglobin levels. The model was significant (R^2^ = 0.74, p < 0.05), and only RBC-MPs were independently associated with the carotid-radial PWV (p < 0.01).

### RBC-MPs Activate Endothelial Cells and Promote Inflammation Through TLR4

**Figure 4:**
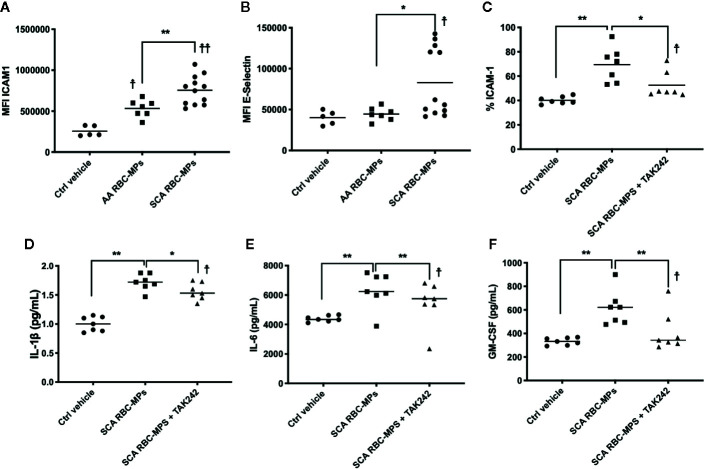
Endothelial cell activation by RBC-MPs and TLR4 inhibition. Impact of RBC-MPs from AA (n = 7) and SCA (n = 12) subjects on ICAM-1 **(A)** and E-Selectin **(B)** expression by HAEC. Impact of TLR4 inhibition (TAK 242) on the effect mediated by SCA RBC-MPs (n = 7) on ICAM-1 expression by HAEC **(C)**. Effects of SCA RBC-MPs incubation on IL-1β **(D)**, IL-6 **(E)** as well as GM-CSF **(F)** production by HAEC, with or without TAK 242. *p < 0.05, **p < 0.01, ^☨^p < 0.05 vs ctrl vehicle, ^☨☨^p < 0.01 vs ctrl vehicle. Statistical comparisons were achieved with Friedmann test followed by Dunn’s test.

To better understand the relationship between RBC-MPs and macrovascular dysfunction, we analyzed the effects of RBC-MPs isolated from SCA and AA blood on the expression of ICAM-1 and E-selectin in HAEC. Incubation of plasma RBC-MPs with HAEC revealed that MPs from SCA patients significantly increased ICAM-1 (p < 0.01) and E-selectin (p < 0.05) expression compared to AA MPs (**Figures 4A, B**, respectively). The large amount of heme transported by RBC-MPs in SCA has been suspected to play a key role in endothelial dysfunction (19, 39). Because heme is an erythrocytic danger-associated molecular pattern (eDAMP) molecule, we further tested the effects of SCA RBC-MPs on ICAM-1 expression in HAEC in the presence or absence of TAK-242, a TLR4 inhibitor. We observed a decrease in the ICAM-1 expression induced by SCA RBC-MPs when inhibiting TLR4 (p < 0.05), although ICAM-1 expression was still higher than in the control condition (**Figure 4C**). Furthermore, the use of TAK-242 decreased the production of IL-1β, IL-6, and GM-CSF by HAEC in comparison to the condition in which HAEC were incubated with RBC-MPs without this inhibitor, but the concentrations of these molecules in the supernatant were still greater than in the control condition (**Figures 4D–F** p < 0.05 for all, 18).

## Discussion

Our study reports for the first time that 1) oxidative stress and NO modulate eryptosis in SCA, 2) oxidative stress increases the release of RBC-MPs in SCA and 3) RBC-MPs directly isolated from SCA blood partly modulate the inflammatory phenotype of endothelial cells through TLR4 activation. In addition, our study confirmed that increased levels of circulating RBC-MPs are associated with increased arterial stiffness in SCA (40).

As previously demonstrated, our results showed higher levels of eryptosis in SCA patients compared to healthy individuals (9, 10, 41, 42). Indeed, we demonstrated that SCA RBCs had higher Ca^2+^ content and increased PS exposure compared to RBC from healthy subjects. Increased PS exposure (1.5% in SCA compared to 0.1% in AA, i.e. a 15-fold increase) could promote RBCs adhesion to the vascular wall and activate coagulation, hence contributing to vaso-occlusive complications (41, 43). *In vitro* studies performed on healthy RBCs have suggested that oxidative stress could trigger eryptosis (6). Several mechanisms contribute to the increased levels of ROS in both plasma and RBCs in SCA (13, 44, 45). We detected higher levels of ROS in RBCs from SCA patients compared to healthy RBCs, and a positive association between RBC ROS level and RBC-PS exposure. We also observed higher glucose uptake in RBCs from patients with SCA compared to healthy RBCs, suggesting an increased metabolic demand and/or an energy depletion; conditions known to trigger eryptosis (46). Several proteins regulate RBC hydration state (47), Ca^2+^ concentration (48) and membrane asymmetry (49) in an energy dependent manner. Therefore, ATP depletion could be involved in eryptosis. Unfortunately, we were not able to assess the impact of hydroxyurea therapy on eryptosis markers as the majority of the cohort was under this treatment. Indeed, hydroxyurea could affect RBC physiology by its effects on oxidative stress and its NO donor properties (50).

Challenging SCA RBCs with antioxidant and pro-oxidant molecules demonstrated that oxidative stress could play an important role in eryptosis. Cumene hydroperoxide promoted eryptosis, while NAC, an antioxidant, limited this phenomenon by improving RBC deformability (+25%), and decreasing ROS (−33.5%), Ca^2+^ (−11.3%) and PS exposure levels (−40%). The increase of RBC deformability observed with NAC could be particularly relevant in the pathophysiological context of SCA, since the loss of deformability is a major contributor of several acute and chronic complications (51, 52). Clinical trials performed in SCA patients showed that NAC limits oxidative stress, hemolysis and RBC dehydration (53, 54). However, the recent study of Sins et al. (55) failed to clearly detect any clinical impact of NAC treatment in SCA. One possible explanation of this disappointing result could be that adherence to NAC treatment was very low in this study. Another explanation for the failure of NAC to provide a significant clinical effect could be its low bioavailability and its hydrophobicity. For this reason it has been suggested that N-Acetylcysteine amides could be more efficient (56). Interestingly, the findings of Sins et al. showed that rates of vaso-occlusive crisis (VOC) were decreased in adherent patients (55). NAC is a precursor of reduced glutathione (GSH), an endogenous antioxidant, and GSH has been shown to be reduced in RBCs of SCA patients (57, 58). Therefore, our results suggest that GSH depletion could be involved in the enhanced eryptosis found in SCA by increasing levels of RBC-ROS. ROS are suspected to activate Ca^2+^ permeable unselective cation channels with subsequent Ca^2+^ entry (6). Increased Ca^2+^ concentration promotes membrane scrambling by inhibiting flippase and activating scramblase and floppase, which disrupt membrane asymmetry, and results in the exposure of PS on the outer leaflet of the membrane (49). Furthermore, the influx of Ca^2+^ into RBCs may activate proteases, like caspase 3 and calpains, that alter membrane and cytoskeleton interactions, leading to membrane blebbing and the release of MPs (59). In the present study, we showed that Cumene hydroperoxide increased Ca^2+^ level in SCA RBCs which could explain the greater RBC-MPs release induced by this oxidative molecule. On the opposite, NAC treatment decreased SCA RBCs Ca^2+^ level which could have contributed to the decrease of RBC-MPs release observed. However, it is unknown whether the MPs production modulation was directly related to oxidative stress process or Ca^2+^ influx.

We also found that the concentrations of RBC-MPs were higher in patients with SCA than in healthy individuals, in accordance with previous studies (14, 15). The positive associations found between AOPP or MDA and the amount of circulating RBC-MPs suggested that oxidative stress would likely play a role in the release of MPs by SCA RBCs. Enhanced oxidative stress during VOC is also suspected to promote RBC-MPs release (16). Indeed, Hierso et al. suggested that exacerbation of oxidative stress during VOC may induce the recruitment of oxidized band 3 in membrane aggregates which could lead to RBC-MPs release (16). It has also been shown in SCD mice model that hemoglobin oxidation and subsequent β-globin posttranslational modifications, including the irreversible oxidation of βCys93 and the ubiquitination of βLys96 and βLys145, were related to band 3 clustering and RBC-MPs emission (60).

NO bioavailability is decreased in SCA (38). It has been suggested that NO modulates eryptosis in healthy RBCs. Nikolay et al. (7) showed that NO could limit PS externalization induced by Ca^2+^ ionophore in healthy RBCs, but no study has tested whether this is also the case in SCA RBCs. Our results showed that incubation of SCA RBCs with SNP effectively increased intracellular NO metabolites. SNP decreased slightly but significantly PS exposure and ROS level and had no effect on Ca^2+^ content. This latter result suggests that the rupture of membrane asymmetry may not be uniquely driven by Ca^2+^ influx, and could explain why no decrease in the concentration of RBC-MPs was observed in the SNP experiments. It has been observed that ROS may directly activate scramblase activity and promote PS exposure (61). Further studies need to be carried out to better understand the mechanism by which SNP can decrease levels of ROS. However, Ca^2+^ can inhibit thioredoxin, an antioxidant and antiapoptotic enzyme. NO has been shown to protect thioredoxin activity by S-nitrosylation of the protein (62), which could explain the decrease in levels of ROS and PS exposure in samples treated with SNP. The increased RBC deformability after incubation with SNP could be the consequence of the lower levels of RBC-ROS, since increasing RBC oxidative stress impairs RBC rheological properties (28). Moreover, the increased RBC β-Spectrin S-nitrosylation caused by SNP could have participated in the improvement of RBC deformability (28). Besides, inhibition of RBC NOS using L-NIO did not affect RBC nitrite content, deformability and eryptosis markers. These results could indicate that RBC NOS was not activated in basal condition in our cohort. We previously showed that RBC-NOS in SCA patients under HU treatment was less activated compared to AA and SCA patients without HU, because of the NO donor properties of the molecule. In the current study, the majority of the patients was under HU treatment which could explain why no effect of L-NIO and only a slight effect of SNP were observed. Indeed, patients under HU treatment had already high level of NO metabolites in RBC which could have limited the potential effects of SNP (50).

Vascular function has been shown to be altered in SCA in both the micro- (21, 63, 64) and macro-circulation (21, 65). It has been suggested that RBC-MPs could participate in the alterations of vascular function in SCA. Camus et al. showed that RBC-MPs generated *ex vivo* induced renal arterial vaso-occlusion in a murine model of SCA, and compromised vasodilation in isolated micro-vessels (66). These effects were mediated by the PS exposed on MPs, which is a common feature of all sub-cellular types of MPs. In 2015, the same group showed that heme-laden RBC-MPs generated *ex vivo* could transfer heme to the vascular endothelium and mediate *in vitro* oxidative stress and apoptosis of endothelial cells (19). However, the MPs used by Camus et al. were generated *ex vivo* by shearing sickle RBCs in a high viscous buffer (30 cP; i.e., 25-fold the viscosity of plasma) with thrombospondin-1 at 1500 s^−1^ (i.e., a very high shear rate that may occur in arterial stenosis or artificial devices). These conditions clearly do not recapitulate those leading to the *in vivo* release of RBC-MPs, and the characteristics of the *in vitro* produced RBC-MPs could be very different from RBC-MPs isolated directly from sickle patients with various clinical histories. Our study revealed an independent association between the concentration of circulating RBC-MPs and arterial stiffness, a marker of macrovascular dysfunction. Tantawy et al. (40) previously found a correlation between RBC-MPs concentration and aortic stiffness, evaluated by echocardiography, in SCA children. However, the direct impact of SCA RBC-MPs on endothelial cells from the macrocirculation has never been investigated. Compared to AA RBC-MPs, RBC-MPs from SCA patients increased the expression of adhesion molecules (ICAM-1 and E-selectin) in human aortic endothelial cells, indicating higher cell activation. These findings are in agreement with a recent study showing an impact of SCA RBC-MPs on microcirculation endothelial cells phenotype (67). ICAM-1 and E-selectin are implicated in circulating cell interactions with the endothelium, which contribute to vascular dysfunction (68). We further showed that inhibition of TLR4 partially decreased the deleterious consequences of MPs on the endothelial cells by limiting ICAM-1 expression and the production of the pro-inflammatory cytokines IL-1β and IL-6. Indeed, RBC-MPs would be able to activate TLR4 and promote inflammasome activation. This mechanism would lead to endothelial cell activation, cell adhesion, and a pro-inflammatory environment that supports vascular dysfunction. IL-1β and IL-6 can activate platelets and leukocytes, promote their adhesiveness (55), and damage the endothelium itself (68). IL-1β has been associated with stroke in SCA (69), and it has been suggested that IL-6 could be related to the development of pulmonary hypertension in SCA children (70). TLR4 inhibition only partially limited the effects mediated by SCA RBC-MPs, which suggests that other mechanisms could be involved in the detrimental consequences of SCA RBC-MPs on endothelial cells. Garnier et al. recently showed that the deleterious effects of SCA MPs on endothelial cells were mediated by the PS exposure on their surface (67). Evaluating the specific phenotype of RBC-MPs from SCA patients could help in better understanding the mechanisms involved in their deleterious effects on endothelial function.

### Limitations

Due to logistical reasons (i.e. limited quantity of collected blood and purified RBC-MPs, and necessity to work on fresh blood sample), *in vivo* modulation experiments have only been performed in a sub-set of the total population recruited. Further studies involving larger cohort are required to fully evaluate the mechanisms at the origin of eryptosis in SCA and the consequences of RBC-MPs on endothelial cells. Besides, in this study, vascular function has been evaluated by pulse wave velocity only. Studies investigating vascular reactivity, and more particularly endothelium-dependent macro- and microvascular reactivity, would help in understanding more deeply the link between vascular dysfunction and RBC-MPs in SCA and could increase the biological relevance of our findings.

## Conclusion

Together, our results suggest that RBC-MPs, that are released during enhanced eryptosis, could play a crucial role in macrovascular dysfunction in SCA patients, and that oxidative stress would modulate eryptosis and RBC-MPs release. RBC-MPs could exert deleterious properties on endothelial cells of the macrocirculation, partly through the activation of TLR4, promoting the expression of adhesion molecules and cytokine production, which may contribute to vascular dysfunction. Further investigations are required to identify the specificity of SCA RBC-MPs at the origin of TLR4 activation, but this study opens new perspectives to understand the underlying mechanisms of vascular dysfunction in SCA. It also points toward new therapeutic targets focusing on preventing eryptosis and/or TLR4 activation in SCA.

## Data Availability Statement

The raw data supporting the conclusions of this article will be made available by the authors, without undue reservation.

## Ethics Statement

The studies involving human participants were reviewed and approved by The CPP Sud-Est IV (Lyon, France, L16-47) and the CPP Sud/Ouest Outre Mer III (Bordeaux, France, 2012‐A00701‐42). Written informed consent to participate in this study was provided by the participants’ legal guardian/next of kin.

## Author Contributions

All authors approved the final version of the manuscript. EN, MaR, MG and PC designed the protocol, performed research, analyzed the data, and wrote the manuscript. NG, YG, SS, CM, CR, PJ, ES, and M-DH-D performed research and edited the manuscript. SA-J and BT designed the protocol and performed research. MG designed the protocol and edited the manuscript. MéR performed research. AG, YB, NL, and RF provided help to organize the different protocols and edited the manuscript. GC and ME-J provided help to organize the different protocols. All authors contributed to the article and approved the submitted version.

## Funding

The study was funded by DFG grant GR4467/3-1.

## Conflict of Interest

MéR was employed by the company Erytech Pharma (Lyon, France).

The remaining authors declare that the research was conducted in the absence of any commercial or financial relationships that could be construed as a potential conflict of interest. Part of the study was funded by DFG grant GR4467/3-1.
