# Supplementary material for: Association Between Nitric Oxide, Oxidative Stress, Eryptosis, Red Blood Cell Microparticles, and Vascular Function in Sickle Cell Anemia
Source: Front Immunol. 2020 Nov 4;11:551441. doi: 10.3389/fimmu.2020.551441 (PMC7672038; doi:10.3389/fimmu.2020.551441)
Supplement: Supplementary file 1 [file DataSheet_1.docx]

Supplementary data

**1**


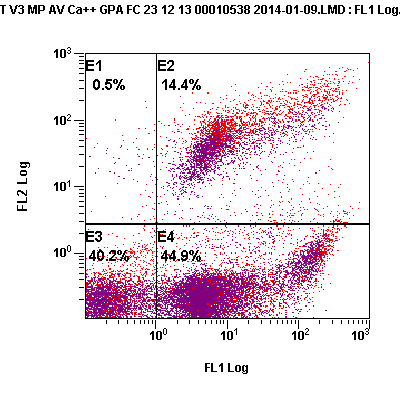

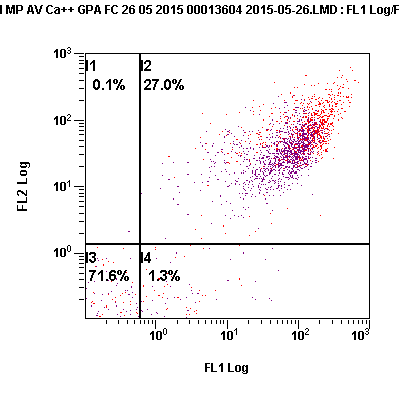

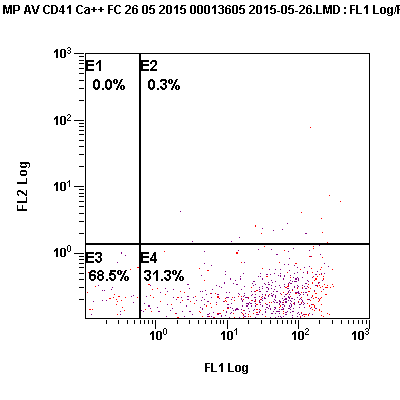

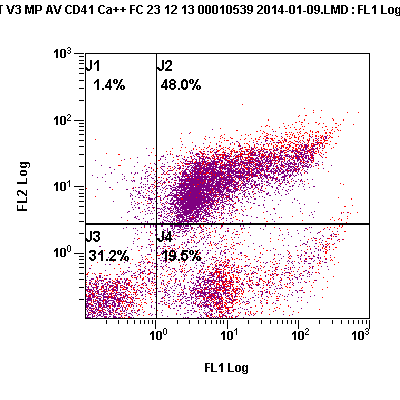


Depleting PLT-MPs

24%

95%

71%

1%

37 561 MP/µL

9 015 MP-GR/µL

10 150 MP/µL

9 642 MP-GR/µL

Labelling AV-FITC/CD235a-PE

Labelling AV-FITC/CD41-PE

Before purification

After purification

Annexin V-FITC

Annexin V-FITC

Annexin V-FITC

Annexin V-FITC

CD41-PE

CD235a-PE

CD235a-PE

CD41-PE

**Supplemental Figure 1**: Representative dot plots showing the percentage of purification of the RBC-MPs used in the endothelial cell’s experiments. After purification, 95% of the MPs obtained originated from RBCs.


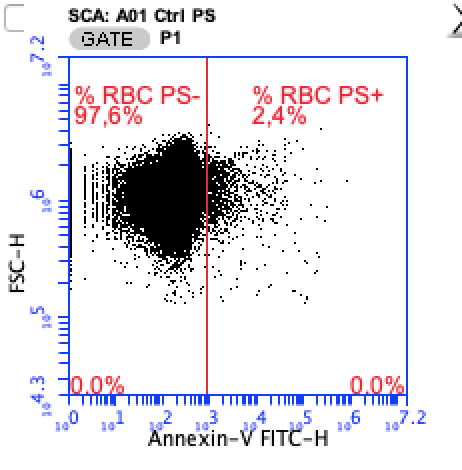

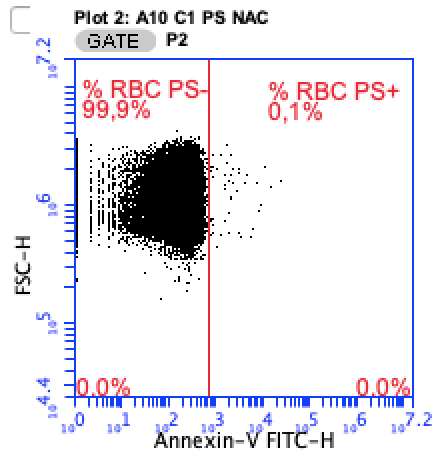


2A

2B

**Supplemental Figure 2**: Representative dot plots showing percentage of RBCs exposing phosphatidylserine at their surface in AA (A) and SCA (B).

**Supplemental Figure 3**: RBC nitrite concentration (A) and β-Spectrin S-nytrosylation in control SCA and SNP condition. *p<0.05 vs ctrl, **<p<0.01 vs ctrl. Conditions were compared using paired t-test.
